# Mucormycosis in critically ill patients: a 9-year multicenter cohort study of clinical features, management, and 90-day outcomes

**DOI:** 10.1186/s13054-026-06227-y

**Published:** 2026-07-31

**Authors:** Frank Bidar, Samy Maamcha, Pauline Tirard-Collet, Jean-Christophe Richard, Arnaud Friggeri, Jean-Luc Fellahi, Matthias Jacquet-Lagrèze, Frederic Aubrun, Laurent Argaud, Frederic Dailler, Fabienne Venet, Florent Valour, Florence Persat, Thomas Rimmelé

**Affiliations:** 1 Department of Anesthesiology and Intensive Care Medicine, University Hospital Louis Pradel, Hospices Civils de Lyon, Bron, France; 2 CarMeN Laboratory, INSERM UMR 1060, Claude Bernard Lyon 1 University, Lyon, France; 3 Department of Anesthesiology and Intensive Care Medicine, Edouard Herriot Hospital, Hospices Civils de Lyon, Lyon, France; 4 Department of Mycology and Parasitology, Institute of Infectious Agents, Croix-Rousse Hospital, Hospices Civils de Lyon, Lyon, France; 5 Medical Intensive Care Unit, Croix-Rousse Hospital, Hospices Civils de Lyon, Lyon, France; 6 Department of Anesthesiology and Intensive Care Medicine, Lyon Sud Hospital, Hospices Civils de Lyon, Pierre-Bénite, France; 7 Department of Anesthesiology and Intensive Care Medicine, Croix-Rousse Hospital, Hospices Civils de Lyon, Lyon, France; 8 Medical Intensive Care Unit, Edouard Herriot Hospital, Hospices Civils de Lyon, Lyon, France; 9 Department of Anesthesiology and Intensive Care Medicine, Pierre Wertheimer Hospital, Hospices Civils de Lyon, Bron, France; 10 Immunology Laboratory, Edouard Herriot Hospital, Hospices Civils de Lyon, Lyon, France; 11 Department of Infectious Diseases, Croix-Rousse Hospital, Hospices Civils de Lyon, Lyon, France

**Keywords:** Mucormycosis, Intensive care unit, Liposomal amphotericin B, Immunosuppression

## Abstract

**Background:**

Mucormycosis is a rare but life-threatening invasive fungal infection that primarily affects immunocompromised patients. Data on its presentation, management, and prognosis in the intensive care unit setting remain scarce. We aimed to evaluate 90-day mortality and to explore clinical, microbiological, therapeutic, and immunological factors associated with outcome in critically ill patients with mucormycosis.

**Methods:**

This retrospective multicenter cohort study included patients diagnosed with mucormycosis and admitted to the ICUs of the *Hospices Civils de Lyon* between January 2014 and July 2023. Patients were identified through mycological records and hospital discharge codes. Univariable analysis and multivariable Cox model were performed to identify factors associated with 90-day mortality.

**Results:**

Among 43 patients included, hematological malignancy and trauma were the most common underlying conditions (26% each), followed by solid organ transplantation (21%), and burns (16%). Cutaneous forms (49%) predominated, especially among trauma and burn patients, while pulmonary and rhinocerebral forms were mostly observed in previously immunocompromised individuals. The number of mucormycosis diagnoses increased nearly threefold over the study period, paralleling the implementation of *Mucorales*-specific polymerase chain reaction. Antifungal therapy was initiated in 39 patients (91%); first-line treatment was liposomal amphotericin B in 32 (74%), at a median maximum dose of 7.5 [3.5–9.4] mg/kg/day, and 12 (28%) received a combination therapy. The 90-day mortality was 47%. In the multivariable Cox model, older age, hematological malignancy, and a higher Sequential Organ Failure Assessment score at diagnosis were independently associated with mortality, while lower liposomal amphotericin B dosing was associated with mortality in univariable analysis. In eight patients without previous immunosuppression and uncontrolled disease, immune monitoring was performed and revealed decreased monocyte HLA-DR expression at 4556 [4194–6936] Ab/c, in favor of injury-induced immunosuppression. Among them, six patients received interferon-gamma as immunotherapy; four responded immunologically and survived.

**Conclusions:**

Mucormycosis in critically ill patients is associated with high mortality, particularly in those with hematological malignancies. Polymerase chain reaction–based diagnostics may allow earlier detection, and immune monitoring could help identify high-risk patients, particularly in trauma and burn settings.

**Supplementary Information:**

The online version contains supplementary material available at https://doi.org/10.1186/s13054-026-06227-y.

## Introduction

Mucormycosis is a rare but life-threatening invasive fungal infection (IFI) caused by filamentous fungi of the *Mucorales* order. It predominantly affects immunocompromised patients, such as those with hematological malignancies, solid organ transplants, uncontrolled diabetes, or those receiving immunosuppressive therapies [1]. Additionally, trauma and burns have emerged as significant risk factors [2, 3]. Mucormycosis can present in various clinical forms, including pulmonary, cutaneous, rhinocerebral, gastrointestinal, and disseminated infections [4]. The disease is also characterized by its angio-invasive nature, leading to rapid tissue necrosis and high mortality rate, especially when diagnosis is delayed and/or treatment inadequate [5]. Indeed, voriconazole, which is the first-line antifungal drug recommended for aspergillosis treatment, the most frequent infection related to filamentous fungi, has no effect on *Mucorales*. Despite being historically considered a rare infection, recent epidemiological studies suggest an increasing incidence, potentially due to a growing immunocompromised population and improved diagnostic methods, particularly the introduction of polymerase chain reaction (PCR) based techniques [6, 7]. Varying rates of mucormycosis have been reported for specific at-risk populations. A multicenter study in France reported a prevalence of one mucormycosis case per 1000 patients with acute lymphocytic leukemia [8] whereas prevalence in solid organ transplant recipients can reach 16% [9].

Although mucormycosis has been well described in immunocompromised populations, few studies have specifically focused on critically ill patients [10, 11]. The available literature has primarily reported on hematological patients, solid organ transplant recipients, and diabetic individuals, without a comprehensive analysis of intensive care unit (ICU)-specific factors. To our knowledge, the immunological profile of ICU patients with mucormycosis has not been specifically investigated, particularly those developing injury-induced immunosuppression [12]. This acquired immune dysfunction, occurring after severe trauma, burns, sepsis, or major surgery, is characterized by alterations in both innate and adaptive immunity, increasing susceptibility to secondary infections [13]. Identifying and characterizing these immune alterations in ICU patients with mucormycosis may inform prognostic assessment and therapeutic strategies.

The primary objective of the present study was to evaluate 90-day mortality in critically ill patients with mucormycosis. Secondary objectives were to identify risk factors, to characterize clinical and microbiological presentations, and to describe management strategies (antifungal treatment, surgical interventions, and immunomodulatory therapies).

## Methods

### Study design and setting

We conducted a retrospective cohort study including patients diagnosed with mucormycosis and admitted to the ten adult ICUs (two surgical ICUs, two medical ICUs, three medico-surgical ICUs, burn unit ICU, neurosurgical ICU and cardiac surgery ICU of the *Hospices Civils de Lyon* (HCL, France) between January 2014 and July 2023. The study was designed to evaluate mortality and prognostic factors associated with mucormycosis in critically ill patients. Patients were identified through two independent data sources: mycological culture results and the French hospital discharge database (*Programme de Médicalisation des Systèmes d’Information*, PMSI). Patients were eligible if they were adults (≥ 18 years) with a diagnosis of mucormycosis either established during the ICU stay or documented before ICU admission when mucormycosis was the reason for ICU admission; no time-since-admission threshold was used to define ICU-related cases. The primary outcome was 90-day all-cause mortality from ICU admission. Attributable mortality was not formally adjudicated.

### Ethical considerations

The ethics committee of the Hospices Civils de Lyon (Comité Scientifique et Ethique des HCL, CSE-HCL – IRB 00013204) approved the study (ref. 23-5475). The research was conducted in accordance with the Declaration of Helsinki. All participants or their family members received an information letter, and none were opposed to the use of their data for research purposes.

### Patient selection and case definition

**Fig. 1:**
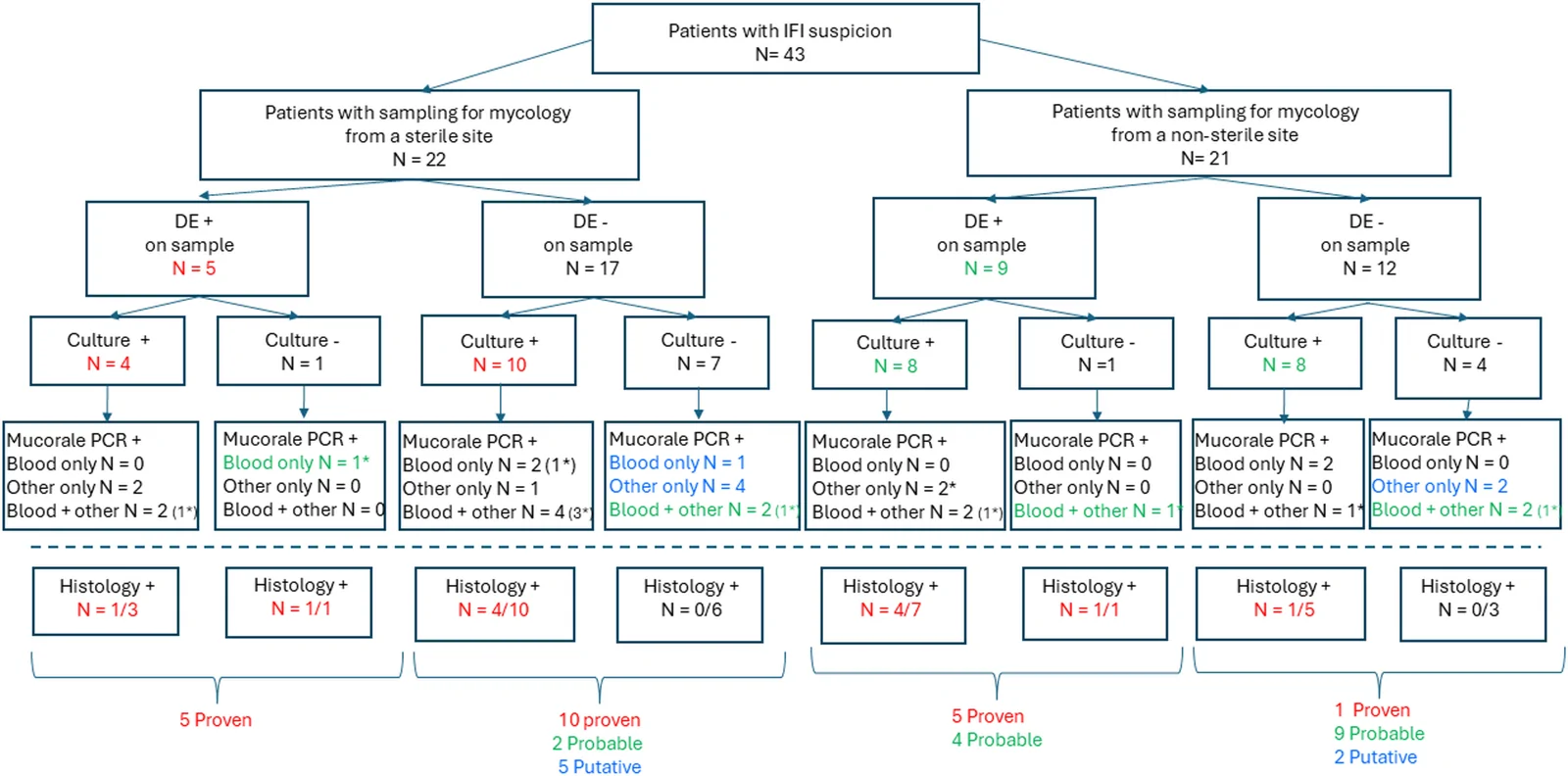
Mucormycosis case classification of patients with infection signs and host factors suggesting an invasive fungal infection, according to biological/histological results adapted from EORTC/MSGERC criteria. Proven cases (red), probable cases (green), putative cases (blue). N= number of patients; DE direct examination. *More than one positive PCR in blood or in samples. Abbreviations: DE, direct examination; EORTC/MSGERC, European Organization for Research and Treatment of Cancer / Mycoses Study Group Education and Research Consortium; PCR, polymerase chain reaction

All patients with a clinical suspicion of invasive fungal infection and a mycology detection of *Mucorales* or a retained diagnosis in the PMSI database were screened. Cases were classified based on the European Organization for Research and Treatment of Cancer/Invasive Fungal Infections Cooperative Group; National Institute of Allergy and Infectious Diseases Mycoses Study Group (EORTC/MSGERC) criteria [14], adapted for mucormycosis, as described by Gouzien et al. [15]. Sample direct examinations were used to categorize the patients (Fig. 1). Direct examinations were considered positive in the following cases: (i) *Mucorales* mycelia were identified and their presence confirmed by culture or PCR or (ii) when undetermined mycelia were detected associated with pure *Mucorales* culture or a sterile culture with positive *Mucorales* PCR directly from the sample. *Mucorales* PCR results were also considered to help in definition of probable cases with at least two positive PCR results (similarly to invasive aspergillosis definition of EORTC 2019) and putative patients with only one positive PCR-based techniques (as defined in the study of Gouzien et al. [15]). PCR-based techniques were used in the present study from 2018 in blood and 2019 in tissues. Mucormycosis colonization was defined as detection of Mucorales by PCR or culture in a non-sterile sample (e.g., respiratory secretions, skin/wound swab) without compatible clinical, biological, or radiological signs of invasive infection. All cases were reviewed in a multidisciplinary discussion involving the mycology and infectious diseases teams; only cases retained as invasive after this review were included in the cohort, while colonized patients were excluded. Redundant cases between databases were excluded.

### Data collection and variables

Clinical, laboratory, and microbiological data were extracted from electronic medical records. Collected variables included demographic (age, sex, comorbidities including hematological malignancy, diabetes, solid organ transplant, trauma, burns, or other causes of immunosuppression), ICU admission characteristics (reason for ICU admission, severity scores, requirement for organ support), presence of acute kidney injury (AKI) defined by Kidney Disease Improving Global Outcomes (KDIGO) definition [16], diagnosis and site of infection, diagnostic method (histology, microscopic direct examination, culture, PCR followed by Sanger sequencing on cultures for species identification and mucorale specific PCR on blood and/or tissue samples), treatment strategies (antifungal therapy, surgical management, and immunomodulatory therapies) and outcomes (30-day and 90-day mortality from ICU admission, ICU length of stay, and readmission).

### PCR methodology

Two complementary molecular approaches were used during the study period (January 2014–July 2023). Genus-targeted Mucorales PCR was performed on plasma and BAL samples. Until March 2023, the in-house quantitative PCR assay developed by Millon et al. [17] was used. This assay combines three separate real-time PCRs targeting different Mucorales genera: one targeting *Mucor*/*Rhizopus*, one targeting *Lichtheimia*, and one targeting *Rhizomucor*; other Mucorales such as *Cunninghamella* are not detected. From late March 2023, this assay was replaced by the commercial MycoGENIE^®^ (Ademtech, Pessac, France) real-time PCR kit targeting the Mucorales order without any further information about the genus or species. No predefined cycle-threshold (Ct) cut-off was applied for Mucorales PCR: positivity was defined by the presence of a specific amplification signal distinguishable from background noise, and Ct values were not reported. The validity of each PCR run was assessed using both negative and positive controls. An internal control was included in each DNA to detect potential PCR inhibition.

In parallel, panfungal PCR was performed on tissue specimens (e.g., sinus, surgical biopsies) when sufficient material was available. Sanger sequencing of the amplicon yielded a species-level identification (a more specific but less sensitive approach), without Ct reporting.

To minimize the risk of contamination, all sample handling procedures were performed in a Class II biosafety cabinet by qualified laboratory personnel, and PCR processing was conducted in dedicated rooms according to a unidirectional workflow to prevent amplicon carryover contamination.

### Immunological assessment

For a subset of patients with no previous history of immunodepression, immune status was assessed using monocyte HLA-DR expression (mHLA-DR) quantification as a marker of immune function [13]. mHLA-DR was not routinely measured in all critically ill patients. It was assessed selectively in patients without prior immunosuppression presenting with uncontrolled mucormycosis, defined as persistent or progressive infection (clinical, microbiological, and/or radiological) despite adequate antifungal therapy and when feasible, with surgical source control. The aim was to evaluate the contribution of injury-induced immunosuppression to disease progression in patients in whom hematological/iatrogenic immunosuppression could not explain treatment failure. The number of HLA-DR molecules per monocyte was determined using the BD Quantibrite Anti-HLA-DR/Anti-Monocyte standardized method (Becton Dickinson, Franklin Lakes, NJ, USA) as previously described [18]. In healthy adults, mHLA-DR values typically range from 13,500 to 45,000 Ab/c [13]. Values below 8,000 Ab/c have been repeatedly associated with ICU-acquired immunosuppression and an increased risk of secondary infections and adverse outcomes in critically ill patients with sepsis, severe trauma, extensive burns, and major surgery [13, 19, 20]. Accordingly, the 8,000 Ab/c threshold was selected a priori, based on this published literature, and was not derived from a data-driven analysis of our cohort. Immunotherapy with interferon-gamma (IFN-γ) was administered on a compassionate-use basis in selected patients with (1) an uncontrolled initial infection despite adequate antifungal therapy and/or interventional procedures; (2) a low level of mHLA-DR expression (< 8000 Ab/c); (3) multidisciplinary decision involving intensivists, immunologists, infectious disease specialists, and mycologists. Before IFN-γ treatment (100 µg per subcutaneous injection for 5 days and stopped if mHLA-DR rose above 20 000 Ab/c), informed consent was obtained for each individual or from the closest relative [20]. A clear explanation of the potential risks and benefits of administering the drug as a compassionate treatment was provided, according to French ethics legislation.

### Statistical analysis

Continuous variables were expressed as medians [interquartile ranges] or means ± standard deviations and compared using the Mann-Whitney U test or Student’s t-test, as appropriate. Categorical variables were presented as counts (percentages) and compared using the chi-square or Fisher’s exact test. Survival analyses were performed using Kaplan-Meier curves with log-rank tests. Cox proportional hazards models were used for multivariable analysis. Three pre-specified covariates were included: age, SOFA score at diagnosis, and hematological malignancy. L-AmB maximal dosing was not retained in the multivariable Cox model to preserve an acceptable event-per-variable ratio. The proportional-hazards assumption was tested using scaled Schoenfeld residuals. Analyses were performed on complete cases; no imputation was used for missing values. All time-to-event analyses were performed from the date of ICU admission, with patients censored at 90 days. All statistical analyses were performed with R software v4.3.1. All reported p-values are two-sided. *P* < 0.05 was considered statistically significant.

## Results

### Diagnosis of mucormycosis

Between January 2014 and July 2023, 48 patients were identified with positive *Mucorales* culture, and 22 patients had a diagnosis of mucormycosis retained in the French PMSI discharge database. After eliminating patients with colonization without infection and duplicate cases identified in both databases, a total of 43 patients were included in the study. During the same period, 219,121 adult admissions were recorded across the ten participating ICUs, corresponding to a cumulative incidence of mucormycosis of 0.20 per 1,000 ICU admissions. Patients were classified according to the EORTC/MSGERC criteria as follows: 21 proven (49%), 15 probable (35%), and 7 putative cases (16%). The classification of clinically suspected patients according to diagnostic methods is reported in Fig. 1. All cases, and particularly the 7 putative cases, were retained as invasive mucormycosis after multidisciplinary review involving intensivists, mycologists and infectious-diseases specialists. The detailed clinical, radiological and microbiological characteristics of the seven putative cases — including specimen sterility — are presented in Supplementary Table 1. The number of patients with a diagnosis of mucormycosis increased over time as shown in Supplementary Fig. 1.

### Patients’ characteristics

Patients’ characteristics are summarized in Table 1. The median age was 51 [42–64] years and 29 (67%) were men. Underlying conditions were heterogeneous, with hematological malignancy (*n* = 11, 26%), trauma (*n* = 11, 26%), solid organ transplantation (*n* = 9, 21%), diabetes mellitus (*n* = 9, 21%), burns (*n* = 7, 16%), other causes of immunosuppression (*n* = 2, 5%), and intravenous drug use (*n* = 2, 5%); two patients had no identified risk factor. Detailed underlying conditions of patients with previous immunosuppression and immunosuppressive therapies are provided in Supplementary Table 2. Median albumin at admission was 20 g/L [18–22]. Severe hypoalbuminemia, defined as albumin ≤ 25 g/L was observed in 79%. Albumin did not significantly differ between survivors (median 19 g/L [17–22], *n* = 14) and non-survivors (21 g/L [19–27], *n* = 10; *p* = 0.23).

**Table 1:** Patients’ characteristics in survivors and non-survivors at day 90 post intensive care unit admission

	All (*n* = 43)	Survivors (*n* = 23)	Non-survivors (*n* = 20)	*p*
**Demographic**				
Sex, Male, n (%)	29 (67)	19 (83)	10 (50)	**0.048**
Age, years, median [IQR]	51 [42–64]	44 [38–58]	60 [49–69]	**0.014**
BMI, kg/m², median [IQR]	26 [23–29]	26 [23–29]	28 [23–31]	0.2
**Risk factors**,** n (%)**				
Hematological malignancy	11 (26)	1 (4.3)	10 (50)	**0.001**
Solid organ transplant	9 (21)	6 (26)	3 (15)	**0.467**
Liver	2 (4.7)	2 (8.7)	0 (0)	
Pulmonary	4 (9)	3 (13)	1 (5)	
Kidney	2 (4.7)	0 (0)	2 (10)	
Kidney and pancreas	1 (2.3)	1 (4.3)	0 (0)	
Trauma	11 (26)	10 (44)	1 (5)	**0.005**
Burns	7 (16)	4 (17)	3 (15)	1
Inflammatory disease	2 (4.7)	1 (4.3)	1 (5)	1
Solid tumor	3 (7)	0 (0)	3 (15)	0.092
Immunosuppressive therapy	18 (42)	7 (30)	11 (55)	0.13
Systemic corticotherapy	15 (35)	5 (22)	10 (50)	0.064
Diabetes mellitus	9 (21)	6 (26)	3 (15)	0.467
Previous antifungal therapy	16 (37)	7 (30)	9 (45)	0.361
**Severity scores**,** median [IQR]**				
SAPS2	56 [45–65]	57 [41–65]	54 [51–68]	0.5
SOFA score at admission	9 [6–12]	10 [4.5–11]	8.5 [6–12]	0.46
SOFA score at diagnosis	7 [4–11]	5 [2–9]	8 [6–12]	0.06
**Laboratory values at admission**,** median [IQR]**				
Leucocytes (10^9^/L)	9.1 [3.6–17]	9.6 [5.6–17]	5.9 [1.5–16]	0.17
Neutrophils (10^9^/L)	7.7 [2.6–13]	8.1 [4.1–14]	5.3 [0.8–13]	0.16
Lymphocytes (10^9^/L)	0.9 [0.2–1.7]	1.3 [0.73–1.9]	0.7 [0.15–1.05]	**0.042**
Monocytes (10^9^/L)	0.5 [0.2–1.2]	0.9 [0.3–1.2]	0.3 [0.1–0.6]	0.07
Creatinine (µmol/L)	119 [80–180]	130 [92–181]	101 [76–175]	0.46
Hemoglobin (g/L)	89 [78–115]	90 [79–133]	86 [78–105]	0.29
Bilirubin (µmol/L)	19 [9–28]	10 [7–20]	22 [11–40]	0.08
Albumin (g/L)	20 [18–22]	19 [17–22]	21 [19–27]	0.23
**Organ failures during ICU stay**,** n (%)**				
Vasopressors	34 (79)	17 (74)	17 (85)	0.467
Renal replacement therapy	25 (58)	12 (52)	13 (65)	0.537
Invasive mechanical ventilation	36 (84)	18 (78)	18 (90)	0.42
**Outcome**				
Mortality at day 30 from ICU admission, n (%)	17 (40)	0 (0)	17 (85)	< 0.001
ICU stay, days, median [IQR]	24 [10–52]	30 [6.8–91]	23 [14–31]	**0.20**
Readmission to ICU for mucormycosis, n (%)	7 (16)	4 (17)	3 (15)	**1**

BMI, body mass index; ICU, intensive care unit; IQR, interquartile range; SAPS2, Simplified Acute Physiology Score 2; SOFA, Sequential Organ Failure Assessment. Significant p values (p < 0.05) are shown in bold

### Clinical manifestations of mucormycosis

Infection was primarily localized to the following sites: 21 (49%) were cutaneous forms, 12 (28%) were pulmonary, 6 (14%) were rhinocerebral, and 1 (2.3%) was abdominal. Overall, 2 (4.7%) patients presented with disseminated mucormycosis (cutaneous and pulmonary; abdominal and rhinocerebral). One patient had only one positive blood PCR, without any obvious entry site, in a context of pulmonary transplantation. The sites of infections are reported in Supplementary Table 3.

Overall, the main sites of infection were associated with the patients’ underlying conditions. Among pulmonary forms, 11/12 (92%) had previous immunosuppression (solid organ transplant or hematological malignancy). Among rhinocerebral forms, 6 (100%) had diabetes mellitus. Cutaneous forms were mainly related to trauma (11/21, 52%) and/or burns (7/21, 33%); 3/21 (14%) occurred in patients with hematological malignancy and 3/21 (14%) followed bacterial necrotizing fasciitis.

### Microbiological diagnosis

Across the 43 patients, mycological sampling was performed from sterile sites in 22 (51%) and from non-sterile sites in 21 (49%). Direct examination was positive in 14 patients (33%) and culture was positive in 30 (70%); the detailed diagnostic pathway is shown in Fig. 1. *Mucorales* were identified by culture or PCR in 35 cases (81%): *Rhizopus* spp. in 12 (34%, including *R. arrhizus* in 7), *Lichtheimia* spp. in 9 (26%), *Mucor* spp. in 7 (20%, mostly *M. circinelloides*), and *Rhizomucor* spp. in 7 (20%). In the remaining 8 cases (19%), diagnosis relied solely on *Mucorales* PCR. *Mucorales* PCR was positive in 13 patients, 8 of whom (62%) were tested between 2022 and 2023. Fungal coinfection was identified in 15 patients (35%) during the ICU stay, including probable *Fusarium* spp. coinfection in four burn patients.

### Antifungal treatment

The median delay between ICU admission and mucormycosis diagnosis was 1 day [IQR 0–14], Antifungal therapy was initiated in 39 patients (91%); two were not treated because of end-of-life decisions and two had a post-mortem diagnosis. First-line therapy was liposomal amphotericin B (L-AmB) in 32 patients (74%). The median initial L-AmB dose was 5 [3.5–8.4] mg/kg/day, with a median maximum dose during ICU stay of 7.5 [3.5–9.4] mg/kg/day. The median delay between diagnosis and L-AmB initiation was 2 days [IQR 0–6], and the median delay between ICU admission and the first antifungal was 5 days [IQR 1–16]. None of these delays significantly differed between survivors and non-survivors (all *p* > 0.2). Initial L-AmB dose did not differ between patients without AKI or AKI stage 1 compared to those with AKI stage 2–3 (5.0 [4.6–8.8] vs. 5.4 [4.1–8.1] mg/kg/day, *p* = 0.77), nor did the maximal dose (9.0 [7.5–10.0] vs. 7.4 [4.9–9.0] mg/kg/day respectively, *p* = 0.20). Combination antifungal therapy at some point during the ICU stay was given in 12 patients (28%). Therapeutic strategies are summarized in Table 2. Antifungal therapies administered before mucormycosis diagnosis are reported in Supplementary Table 4.

**Table 2:** Therapeutic strategies in intensive care unit

	Survivors (*n* = 23)	Non-survivors (*n* = 20)	All (*n* = 43)	*p*
Received antifungal therapy, *n* (%)	23 (100)	16 (80)	39 (91)	0.084
First-line antifungal therapy, n (%)				0.16
None	0 (0)	4 (21)	4 (9.3)	
Amphotericin B	0 (0)	1 (5)	1 (2.3)	
Liposomal amphotericin B	19 (83)	13 (65)	32 (74)	
Isavuconazole	2 (8.7)	1 (5)	3 (7)	
Posaconazole	2 (8.7)	1 (5)	3 (7)	
Combination antifungal therapy during ICU stay, n (%)	7 (30)	5 (25)	12 (28)	0.82
Initial liposomal amphotericin B dosing, mg/Kg/day, median [IQR]	7.5 [4.7–9.7]	3.7 [0.8–5.1]	5 [3.5–8.4]	**0.01**
Maximal liposomal amphotericin B dosing, mg/Kg/day, median [IQR]	8.2 [7.2–10]	3.7 [0.8–6.4]	7.5 [3.5–9.4]	**0.01**
Antifungal instillation, n (%)	11 (48)	4 (20)	15 (35)	0.11
Hyperbaric oxygen therapy, n (%)	10 (44)	2 (10)	12 (28)	0.036
Surgery, n (%)	18 (78)	7 (35)	25 (58)	0.011

*Antifungal instillation refers to local administration of an antifungal agent (most often liposomal amphotericin B), either as intra-operative instillation in the surgical field or as topical application on wound dressings

ICU, intensive care unit; IQR, interquartile range. Significant p values (p < 0.05) are shown in bold

### Surgical procedures

Surgery was performed in 25 patients (58%). The proportion varied markedly by infection site: cutaneous 18/21 (86%), rhinocerebral 4/6 (67%, the two non-operated patients had end-of-life decisions), and pulmonary 1/12 (8%); the three non-operated cutaneous cases were considered ineligible because of secondary cutaneous involvement (*n* = 2) or post-mortem diagnosis (*n* = 1). A repeat procedure was required in 21/25 operated patients (84%), with multiple procedures (≥ 3) in 16/25 (64%). Hyperbaric oxygen therapy was used as adjunctive treatment in 12/25 operated patients (48%) (Table 2). In patients with repeated procedures, surgery was planned for clinical failure (progression of necrosis or persistent positive mycology) or for systematic second-look procedures which is standard of care in burn and sometimes trauma management.

### Immune monitoring and immunotherapy

**Fig. 2:**
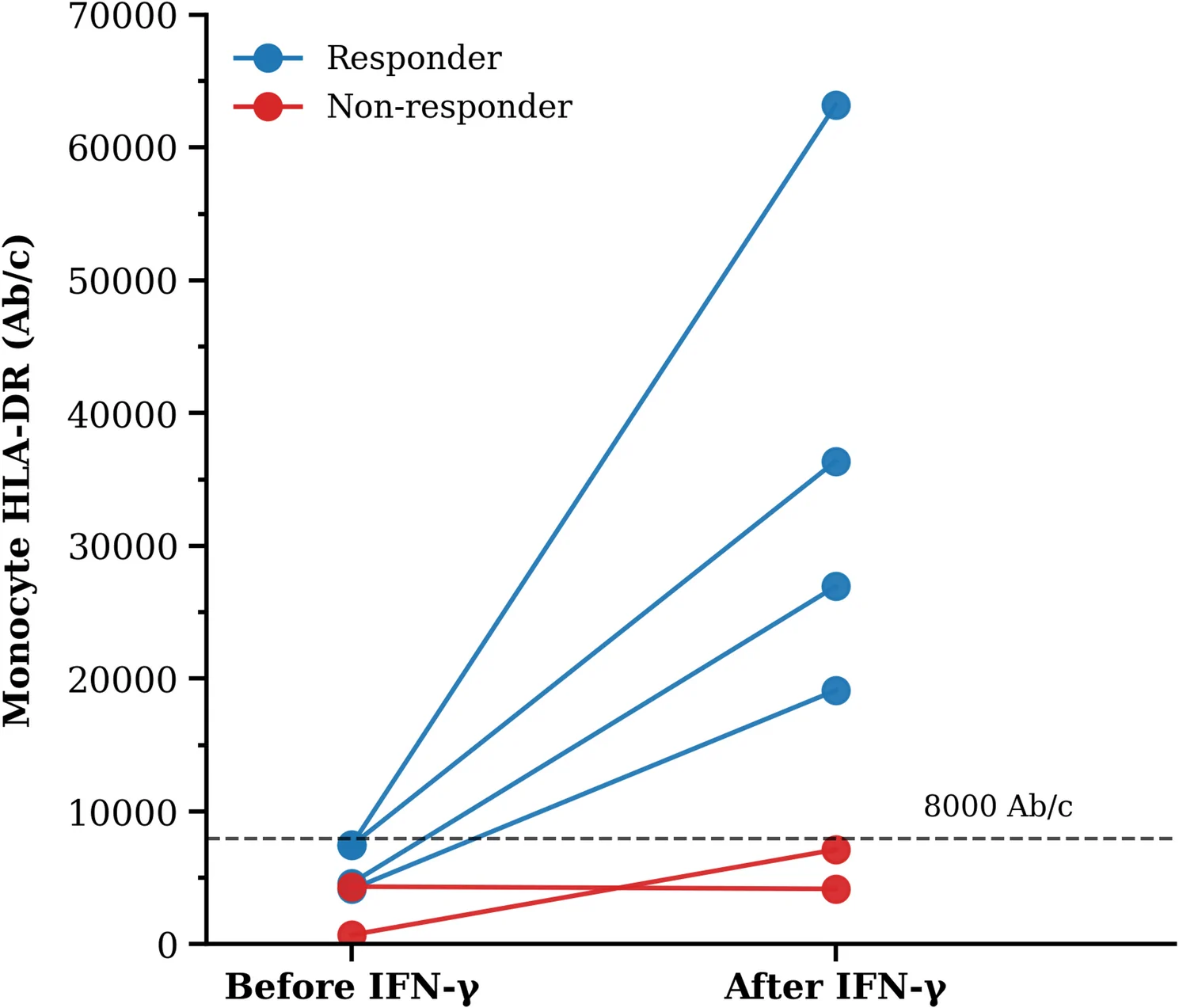
Monocyte HLA-DR (mHLA-DR) expression before and after IFN-γ administration. Each line connects baseline (just before IFN-γ) and post-treatment values for the six patients with available paired measurements. Responders were defined as patients whose mHLA-DR rose above 8000 Ab/c after treatment (dashed line). Abbreviations: Ab/c, antibodies bound per cell; HLA-DR, human leukocyte antigen-DR isotype; IFN-γ, interferon-gamma; mHLA-DR, monocyte HLA-DR

At ICU admission, patients frequently displayed lymphopenia, with a median lymphocyte count of 0.9 [0.2–1.7] ×10⁹/L and a median monocyte count of 0.5 [0.2–1.2] ×10⁹/L (Table 1); both were lower in non-survivors than in survivors (lymphocytes 0.7 [0.15–1.05] vs. 1.3 [0.73–1.9] ×10⁹/L, *p* = 0.042; monocytes 0.3 [0.1–0.6] vs. 0.9 [0.3–1.2] ×10⁹/L, *p* = 0.07). At the time of mucormycosis diagnosis, the median lymphocyte and monocyte counts were 1.1 [0.7–1.6] and 0.6 [0.3–1.0] ×10⁹/L, respectively; lymphocyte counts no longer differed between survivors and non-survivors whereas monocyte counts remained significantly lower in non-survivors (0.4 [0.1–0.7] vs. 0.9 [0.6–1.0] ×10⁹/L, *p* = 0.02). Eighteen patients (42%) did not have any previous history of immunosuppression (i.e. hematological malignancy, organ transplantation, immunosuppressive therapy, or severely decompensated diabetes). These patients were admitted to the ICU in a context of trauma, burns, or necrotizing fasciitis. Immune monitoring via evaluation of mHLA-DR expression was performed in case of uncontrolled disease, so that eight of these patients were monitored during their ICU stay. Their median mHLA-DR values at mucormycosis diagnosis was 4556 [4194–6936] Ab/c and in favor of severe injury-induced immunosuppression. Six of these eight patients received compassionate IFN-γ in a context of low mHLA-DR values and uncontrolled infection. After treatment with IFN-γ, median mHLA-DR expression increased to 23 028 [10121–34017] Ab/c. Individual mHLA-DR values before and after IFN-γ are shown in Fig. 2.

### ICU outcome and mortality

The 90-day mortality rate was 47%, with 40% of patients (17/43) already deceased by day 30; The median ICU length of stay was 24 days [IQR 10–52] in the whole cohort, with no significant difference between survivors (30 days [6.8–91]) and non-survivors (23 [14–31]; *p* = 0.2). Seven patients (16%) were re-admitted to the ICU for mucormycosis-related events, with no difference between groups (4 survivors [17%] vs. 3 non-survivors [15%]; *p* = 1.00). Most patients presented with multiple organ failure, as 36 (84%) were under mechanical ventilation, 34 (79%) under vasopressors, and 25 (58%) required renal replacement therapy during their ICU stay. Several factors were associated with outcomes in univariate analysis, notably age, hematological malignancy, lower lymphocyte levels, and lower dosing of L-AmB were associated with mortality (Tables 1 and 2).

Survival analyses were performed in the 39 treated patients; Kaplan-Meier curves stratified by hematological malignancy and L-AmB dosing are shown in Supplementary Fig. 2. In the multivariable Cox model, SOFA score, age and hematological malignancy remained independently associated with 90-day mortality (Table 3).

**Table 3:** Multivariable analysis with Cox proportional hazards model

Factor	HR [95% CI]	*p*-value
Age, per year	1.07 [1.02 ; 1.12]	0.003
Hematological malignancy	14.7 [3.68 ; 58.5]	< 0.001
SOFA score at diagnosis, per point	1.16 [1.01 ; 1.34]	0.033

CI, confidence interval; HR, hazard ratio; L-AmB, liposomal amphotericin B; SOFA, Sequential Organ Failure Assessment

Among the six patients treated with IFN-γ, four had a positive immunological response (i.e. increase in mHLA-DR above 8000 Ab/c) and all survived. The two remaining patients, classified as non-responders, both died in ICU. The two non-responders had two distinguishing features compared with the responders: a much later mucormycosis diagnosis (median 72 vs. 22 days after ICU admission) and the absence of complete surgical resection at the time of IFN-γ initiation. Notably, baseline SOFA scores at diagnosis were not higher in non-responders.

## Discussion

In this multicenter cohort study, mucormycosis in critically ill patients was associated with high mortality, particularly in patients with hematological malignancy. Compared to previous ICU cohorts [10, 11], our cohort included a higher proportion of trauma and burn patients, who had more favorable outcomes. The implementation of PCR-based diagnostic techniques may have contributed to the identification of additional cases in this cohort. Higher L-AmB doses were associated with improved survival in univariable analysis, supporting the importance of adequate antifungal dosing. We also documented injury-induced immunosuppression in patients with uncontrolled mucormycosis without prior immunodepression, supporting a potential role for immune monitoring in this setting.

The number of mucormycosis diagnoses in our study increased nearly threefold between 2014 and 2023. This rise likely reflects increased awareness in ICUs and the recent availability of *Mucorales*-targeted PCR, which has substantially improved laboratory diagnosis, consistent with a French nationwide study and a recent meta-analysis [15, 21]. In ICU patients, in whom clinical examination may be challenging, *Mucorales* PCR—despite not yet being included in international guidelines—may facilitate earlier diagnosis, given its high sensitivity and specificity. Patients diagnosed before and after PCR implementation may therefore not represent fully comparable populations, and the apparent temporal trend may be better explained by improvements in diagnostic methods rather than by a true epidemiological increase.

The prognosis of mucormycosis is generally poor, with high ICU mortality rates. The lower 90-day mortality observed in our cohort likely reflects the patient mix, with an equal number of trauma patients and patients with hematological malignancy. Hematological malignancy is a well-established risk factor for mucormycosis and is strongly associated with poor outcomes [15, 22]. In the study by Gouzien et al. [15], hematological patients accounted for 65% of mucormycosis cases with a mortality of 64%, although these data were not limited to ICU settings. Another multicenter ICU-specific study reported a mortality of 77% in critically ill patients with hematological malignancy and mucormycosis [11], consistent with our findings in the hematological subgroup. Age, another well-recognized risk factor, was also associated with poorer outcomes in our cohort [22, 23]. The apparent association between surgical management and improved survival in our study should be interpreted considering confounding by infection site. In our cohort, cutaneous disease (predominantly in trauma and burn patients) accounted for most survivors and was virtually always surgically managed, whereas pulmonary and disseminated forms, more frequent in patients with hematological malignancy, were rarely eligible to surgery and were associated with worse prognosis. The univariable association between surgery and survival therefore largely reflects the underlying infection-site distribution and should not be interpreted as evidence of an independent therapeutic effect of surgery.

Prompt initiation of effective therapy is critical in mucormycosis, as treatment delay is associated with mortality [5]. *Mucorales* PCR allows faster diagnosis than culture or histology [17]. A recent study reported a lower mortality rate after implementation of PCR testing [15], likely because PCR allows for a diagnosis four days earlier than other conventional testing [24]. In ICU patients, where clinical signs are non-specific and examination may be limited, this gain may be particularly relevant. In the participating ICUs, local protocols include systematic *Mucorales* PCR on tissues from trauma and burn patients undergoing surgery since 2019. Legrand et al. previously reported detection of circulating *Mucorales* DNA up to 11 days before standard diagnosis in burn patients, allowing earlier antifungal initiation [25]. A multidisciplinary approach involving intensivists, mycologists, infectious disease specialists, and surgeons may further facilitate early diagnosis, especially in trauma and burn patients. Adequate antifungal exposure is a key determinant of outcome in mucormycosis. In our cohort, higher L-AmB doses were associated with lower mortality in univariable analysis, consistent with previous reports. The 2019 European guidelines recommend first-line L-AmB at 5–10 mg/kg/day, with 10 mg/kg/day in case of central nervous system involvement [1, 26]. Higher L-AmB doses have been associated with higher response rates in dose-finding studies [27]. However, acute kidney injury is common in ICU patients and may be worsened by L-AmB. Dosing antifungals in critically ill patients is challenging: pharmacokinetic targets may be missed because of altered volume of distribution, particularly in patients with septic shock, trauma, or burns [28] and because vascular thrombosis and necrosis can limit drug penetration into infected tissues. The association between higher L-AmB dosing and improved survival was only reported in univariable analysis and should be interpreted with caution given several plausible sources of bias. First, immortal-time bias may exist as patients who died early in their ICU course did not have time to undergo dose escalation. In our cohort, the median time from ICU admission to L-AmB initiation was longer in survivors than in non-survivors, this numerically shorter delay in non-survivors argues against a delayed treatment effect but is consistent with immortal-time bias. Second, confounding by indication may occur as patients with AKI, end-of-life decisions, or ineligible for aggressive surgical management (predominantly pulmonary or disseminated forms in patients with hematological malignancy) were more likely to receive lower doses for clinical reasons, while others may have benefited from more aggressive antifungal management. Antifungal combinations were not associated with outcome in our cohort, but this could reflect confounding by indication, as the most severely ill patients tend to receive combination therapy [11].

We also report immune monitoring performed in this specific population. Injury-induced immunosuppression is an acquired state of impaired innate and adaptive immunity that follows a severe initial injury such as sepsis, trauma, extensive burns, or major surgery [12, 29] that can be assessed by several markers including low mHLA-DR expression [30, 31]. In a subgroup of patients without prior immunodepression, we found markedly low mHLA-DR values at mucormycosis diagnosis, consistent with severe injury-induced immunosuppression. Notably, two of these patients had no history of trauma or burns; diagnosis of mucormycosis followed severe necrotizing fasciitis secondary to streptococcal A toxic shock. Beyond mHLA-DR, lower lymphocyte counts at ICU admission, another marker of immunosuppression, were also associated with worse outcomes, in line with prior ICU studies [32]. We also describe the use of compassionate IFN-γ immunotherapy in patients with refractory infection. IFN-γ has shown encouraging results in selected patients with sepsis or invasive fungal infections [20, 33, 34]. However, unselected use of such therapies could have negative adverse events [35]. In our institutions, IFN-γ is administered only after multidisciplinary discussion involving intensivists, immunologists, mycologists, and infectious disease specialists. Among the six patients treated, the four immunological responders all survived, whereas the two patients without immunological response died in ICU. The determinants of non-response in this small descriptive series were the duration of pre-existing injury-induced immunosuppression and inadequate surgical source control rather than acute organ-failure severity at the time of immune intervention. These observations could help guide future administration of such therapies. Whether IFN-γ improves outcome in critically ill patients with mucormycosis remains to be proven and warrants confirmation in dedicated studies. The immunological findings reported here are exploratory and hypothesis-generating as mHLA-DR was assessed only in a highly selected subgroup of patients without prior immunosuppression in whom disease control could not be achieved despite antifungal therapy and surgery, limiting generalizability. The descriptive series of six patients treated with adjunctive IFN-γ on a compassionate basis does not allow any inference of therapeutic efficacy and should not be interpreted as supporting routine clinical use of this strategy.

Beyond classical risk factors, immunometabolic dysregulation is increasingly recognized as a determinant of host susceptibility to mucormycosis and of clinical outcomes. Recent mechanistic work has identified albumin as a regulator of natural host defense against *Mucorales* [36]. In our cohort, albumin at admission was profoundly reduced. The absence of a significant albumin difference between survivors and non-survivors in our subset likely reflects a floor effect, as virtually all evaluable patients were already in the high-risk hypoalbuminemic range. These observations align with the emerging concept that immunometabolic dysregulation contributes to mucormycosis susceptibility and severity in critically ill patients and support the integration of systematic nutritional and metabolic assessment, including serum albumin, into future studies of mucormycosis in the ICU.

Our study has several limitations. First, diagnosis was based solely on PCR in some cases. Although *Mucorales* PCR is not yet included in international guidelines, it is widely used in clinical practice and has demonstrated excellent sensitivity and specificity [24], and may be considered for inclusion in future revisions of the EORTC/MSGERC criteria [21]. PCR-based criteria are not yet universally consensual, particularly for non-sterile samples in trauma and burn patients in whom environmental contamination or transient colonization may not always be excluded. By incorporating Mucorales PCR results into the case definition, our study is exposed to a risk of incorporation bias: PCR contributes both to case ascertainment and to the analyses describing how PCR implementation has improved diagnosis. Findings related to the temporal increase in incidence should therefore be interpreted with caution. All PCR-only cases had a clinical presentation compatible with mucormycosis and were reviewed by a multidisciplinary team. Clarifying the classification of probable and putative cases, particularly the threshold of one versus two positive PCR results, would improve standardization. Second, the retrospective design carries inherent limitations: some ICU mucormycosis cases may have been missed despite screening through both the mycology laboratory and the French hospital discharge database. Third, the heterogeneous population and limited sample size restricted subgroup analyses, results should therefore be interpreted as exploratory. However, given that patients were included from a large number of ICUs with diverse specializations, the study population is representative of the broad variety of critically ill patients who present with mucormycosis. Fourth, detailed pharmacokinetic/pharmacodynamic parameters, including measured 24-hour creatinine clearance, the prevalence of augmented renal clearance, and therapeutic drug monitoring of L-AmB were not available for enough patients to allow a reliable analysis in this retrospective cohort. Finally, immune monitoring was not performed in all patients; implementing this strategy systematically in patients with unfavorable outcomes could help identify those with immune dysfunction.

## Conclusion

Mucormycosis in critically ill patients is associated with high mortality, particularly in those with hematological malignancies. PCR-based diagnostics may allow earlier detection, and immune monitoring could help identify high-risk patients, particularly in trauma, burn, and post-surgical settings. Future studies should focus on optimizing antifungal dosing and earlier diagnosis to improve outcomes in this high-risk population.

## Supplementary Information

Below is the link to the electronic supplementary material.

Supplementary Material 1: Mucormycosis case detection dates of the 43 patients diagnosed between January 2014 and July 2023. The retained dates correspond to the first sample with a positive Mucorales result. Data are presented as the number of cases per year. * implementation of PCR testing in blood in Lyon. ** implementation of PCR testing in tissues in Lyon. Abbreviations: PCR, polymerase chain reaction.

Supplementary Material 2: Kaplan-Meier curves of mortality from ICU admission according to hematological malignancy and liposomal amphotericin B dosing (initial and highest dose). (a) Cumulative mortality according to the presence of a hematological malignancy. (b) Cumulative mortality according to the highest liposomal amphotericin B (L-AmB) daily dose achieved during the ICU stay (<5 vs ≥5 mg/kg/day). (c) Cumulative mortality according to the initial L-AmB daily dose (<5 vs ≥5 mg/kg/day). p-values were calculated from log-rank tests. Abbreviations: ICU, intensive care unit; L-AmB, liposomal amphotericin B.

Supplementary Material 3.

Supplementary Material 4.

Supplementary Material 5.

Supplementary Material 6.

## Data Availability

No datasets were generated or analysed during the current study.

## Abbreviations

Ab/c: Antibodies bound per cell
BMI: Body mass index
CI: Confidence interval
CSE-HCL: Comité Scientifique et Ethique des Hospices Civils de Lyon
DE: Direct examination
EORTC/MSGERC: European Organization for Research and Treatment of Cancer / Mycoses Study Group Education and Research Consortium
HCL: Hospices Civils de Lyon
HR: Hazard ratio
ICU: Intensive care unit
IFI: Invasive fungal infection
IFN-γ: Interferon-gamma
IQR: Interquartile range
IRB: Institutional Review Board
L-AmB: Liposomal amphotericin B
mHLA-DR: Monocyte human leukocyte antigen-DR isotype expression
PCR: Polymerase chain reaction
PMSI: Programme de Médicalisation des Systèmes d’Information
SAPS2: Simplified Acute Physiology Score 2
SOFA: Sequential Organ Failure Assessment
