# Supplementary material for: Mucormycosis in critically ill patients: a 9-year multicenter cohort study of clinical features, management, and 90-day outcomes
Source: Crit Care. 2026 Jul 31;30:542. doi: 10.1186/s13054-026-06227-y (PMC13693191; doi:10.1186/s13054-026-06227-y)
Supplement: Supplementary file 1 [file 13054_2026_6227_MOESM3_ESM.docx]

**Supplementary Table 1. Detailed description of the 7 patients classified as putative mucormycosis cases (positive Mucorales PCR in a local sample only, in the absence of positive blood PCR, culture, direct examination or histology).**

| **Patient** | **Underlying condition** | **Site** | **Imaging at diagnosis** | **Specimen type** | **Sterility** | **PCR result(s)** |
| --- | --- | --- | --- | --- | --- | --- |
| P1 | Hematological malignancy (multiple myeloma + MDS, allo-HSCT, immunosuppressants, corticosteroids) | Pulmonary and disseminated | CT chest: pulmonary nodules / consolidation | BAL | Non-sterile | Local Mucorales PCR positive (BAL); blood PCR negative |
| P2 | Solid organ transplant (lung), immunosuppressants | Pulmonary | CT chest: pulmonary infiltrates | BAL | Non-sterile | Local Mucorales PCR positive (BAL); blood PCR negative |
| P3 | Trauma | Cutaneous (deep soft tissue) | Clinical wound assessment | Muscle biopsy | Sterile | Local Mucorales PCR positive; blood PCR negative |
| P4 | No prior immunosuppression (cutaneous mucormycosis on severe injury) | Cutaneous (abscess) | Clinical assessment; surgical exploration | Abscess (intra-operative) | Sterile | Local Mucorales PCR positive; blood PCR not available |
| P5 | Trauma | Cutaneous (deep soft tissue) | Clinical wound assessment; surgical exploration | Surgical biopsies (intra-operative) | Sterile | Local Mucorales PCR positive; blood PCR negative |
| P6 | Trauma | Cutaneous (deep soft tissue with suppuration) | Clinical wound assessment; surgical exploration | Deep tissue biopsy / suppuration | Sterile | Local Mucorales PCR positive; blood PCR negative |
| P7 | Trauma + burns | Cutaneous | Clinical wound assessment of burn site | Skin biopsy | Non-sterile | Local Mucorales PCR positive (Rhizopus spp); blood PCR negative |

*Distribution of specimen: 4/7 putative cases were diagnosed on a sterile-site biopsy (intra-operative deep cutaneous/muscle biopsy in trauma patients), 2/7 on a non-sterile BAL (pulmonary forms in hematological/SOT patients), and 1/7 on a non-sterile skin biopsy (trauma and burn). Mucorales species identification was successful in 2/7 cases (Lichtheimia spp. in one cutaneous case, Rhizopus spp. in one burn-trauma case); the remaining 5 cases were classified as undetermined (species could not be identified at the molecular level).*

*Sterility refers to the nature of the sampling site: sterile sites (deep tissue/muscle/intra-operative biopsy) versus non-sterile sites (BAL, skin) where environmental contamination or transient colonization can theoretically occur. Imaging descriptions are inferred from the clinical context and require confirmation against individual records.*

***Abbreviations:*** *BAL, bronchoalveolar lavage; CT, computed tomography; HSCT, hematopoietic stem cell transplantation; MDS, myelodysplastic syndrome; MRI, magnetic resonance imaging; PCR, polymerase chain reaction; SOT, solid organ transplantation.*
