# Supplementary material for: Mucormycosis in critically ill patients: a 9-year multicenter cohort study of clinical features, management, and 90-day outcomes
Source: Crit Care. 2026 Jul 31;30:542. doi: 10.1186/s13054-026-06227-y (PMC13693191; doi:10.1186/s13054-026-06227-y)
Supplement: Supplementary file 2 [file 13054_2026_6227_MOESM4_ESM.docx]

**Supplementary Table 3. Sites of infection in** **survivors and non-survivors at day 90 post intensive care unit admission**

|  | Survivors  (n = 23) | Non-survivors  (n = 20) | All  (n = 43) |
| --- | --- | --- | --- |
| Localized, n (%) |  |  |  |
| Cutaneous | 15 (65) | 6 (30) | 21 (49) |
| Abdominal | 0 (0) | 1 (5) | 1 (2.3) |
| Pulmonary | 4 (17) | 8 (40) | 12 (28) |
| Rhinocerebral | 3 (13) | 3 (15) | 6 (14) |
| Disseminated, n (%) | 0 (0) | 2 (10) | 2 (4.7) |
| Isolated blood PCR, n (%) | 1 (4.3) | 0 (0) | 1 (2.3) |

*Abbreviations:* PCR, polymerase chain reaction.
