# Supplementary material for: Mucormycosis in critically ill patients: a 9-year multicenter cohort study of clinical features, management, and 90-day outcomes
Source: Crit Care. 2026 Jul 31;30:542. doi: 10.1186/s13054-026-06227-y (PMC13693191; doi:10.1186/s13054-026-06227-y)
Supplement: Supplementary file 3 [file 13054_2026_6227_MOESM5_ESM.docx]

**Supplementary Table 4. Prior antifungal therapy among the 16 patients exposed before mucormycosis diagnosis.**

| **Patient** | **Underlying condition** | **Antifungal agent(s)** | **Indication** | **Reason** |
| --- | --- | --- | --- | --- |
| P1 | SOT + inflammatory disease | Voriconazole | Curative | Pulmonary and sinus aspergillosis |
| P2 | Trauma | Fluconazole then caspofungin | Curative | Peritonitis C. albicans / C. tropicalis |
| P3 | Hematological malignancy | Fluconazole | Preemptive |  |
| P4 | Hematological malignancy + allo-HSCT | L-AmB + voriconazole | Preemptive |  |
| P5 | SOT (kidney) | Inhaled L-AmB | Prophylactic |  |
| P6 | SOT (lung) | Inhaled amphotericin B | Prophylactic |  |
| P7 | Hematological malignancy + allo-HSCT | Posaconazole | Curative | Pulmonary fusariosis |
| P8 | SOT (lung) | Isavuconazole | Preemptive |  |
| P9 | Hematological malignancy | Voriconazole then caspofungin | Preemptive |  |
| P10 | Burns | Fluconazole | Curative | Candidemia (Candida albicans) |
| P11 | SOT (kidney) | Voriconazole | Curative | Invasive pulmonary aspergillosis |
| P12 | Hematological malignancy | Fluconazole | Curative | Candidemia |
| P13 | Hematological malignancy + allo-HSCT | Posaconazole | Prophylactic |  |
| P14 | Hematological malignancy | Voriconazole + L-AmB (started 3 d pre-ICU) | Preemptive |  |
| P15 | Solid tumor | Fluconazole | Curative | Candida albicans peritonitis |
| P16 | Trauma + burns | Voriconazole | Curative | Skin/soft tissue infection (Aspergillus nidulans/flavus) |

*Curative: treatment of a documented infection. Preemptive: treatment based on a positive microbiological or imaging signal without proven infection. Prophylactic: primary mold-active prophylaxis without any clinical or microbiological signal. Patient identifiers are anonymized*

*Abbreviations: CT, computed tomography; HSCT, hematopoietic stem cell transplantation; L-AmB, liposomal amphotericin B; PCR, polymerase chain reaction; SOT, solid organ transplantation.*
