# Supplementary material for: Mucormycosis in critically ill patients: a 9-year multicenter cohort study of clinical features, management, and 90-day outcomes
Source: Crit Care. 2026 Jul 31;30:542. doi: 10.1186/s13054-026-06227-y (PMC13693191; doi:10.1186/s13054-026-06227-y)
Supplement: Supplementary file 4 [file 13054_2026_6227_MOESM6_ESM.docx]

**Supplementary Table 2. Detailed underlying conditions at the time of mucormycosis diagnosis in the 22 patients with hematological malignancy, solid organ transplant, solid tumor or inflammatory disease**

| Patients | **Underlying condition** | **Specific diagnosis / detail** | **Immunosuppressive therapy at diagnosis** |
| --- | --- | --- | --- |
| H1 | Hematological malignancy | Cutaneous T-cell lymphoma, low grade | Methotrexate, methylprednisolone + cytotoxic chemotherapy |
| H2 | Hematological malignancy | Diffuse large B-cell lymphoma, high grade | Ifosfamide + etoposide corticosteroids |
| H3 | Hematological malignancy | AML M1, allogeneic HSCT | Cyclophosphamide, mycophenolate, ciclosporin |
| H4 | Hematological malignancy | Multiple myeloma + high-risk myelodysplastic syndrome, allogeneic HSCT | Methylprednisolone, ciclosporin, lenalidomide |
| H5 | Hematological malignancy | Suspected myelodysplastic syndrome | None recorded |
| H6 | Hematological malignancy | AML M0, undifferentiated | Dexamethasone |
| H7 | Hematological malignancy | Cerebral high-grade diffuse B-cell lymphoma, autologous HSCT | Intensification chemotherapy (thiotepa, busulfan, mesna, cyclophosphamide) + intrathecal MTX, then autologous HSCT; corticosteroids |
| H8 | Hematological malignancy | Chronic myelomonocytic leukaemia (myeloproliferative syndrome) | Corticosteroids |
| H9 | Hematological malignancy | B-cell ALL, allogeneic HSCT | None recorded at diagnosis |
| H10 | Hematological malignancy | AML M1 | None recorded |
| H11 | Hematological malignancy | High-grade diffuse large B-cell lymphoma | None recorded |
| T1 | Solid organ transplant | Liver transplant | Tacrolimus, hydrocortisone, mycophenolate |
| T2 | Solid organ transplant | Lung transplant | Basiliximab (induction); corticosteroids |
| T3 | Solid organ transplant | Lung transplant | Tacrolimus, mycophenolate, basiliximab, methylprednisolone then prednisone |
| T4 | Solid organ transplant | Lung transplant | Tacrolimus, mycophenolate, corticosteroids |
| T5 | Solid organ transplant | Lung transplant | Mycophenolate, tacrolimus, corticosteroids, everolimus |
| T6 | Solid organ transplant | Liver transplant | Tacrolimus, everolimus |
| T7 | Solid organ transplant | Kidney transplant | Prednisone, tacrolimus, mycophenolate |
| T8 | Solid organ transplant | Kidney–pancreas transplant | Tacrolimus, prednisone 5 mg/day |
| T9 | Solid organ transplant | Kidney transplant | Tacrolimus, mycophenolate, corticosteroids |
| S1 | Solid tumor |  |  |
| O1 | Inflammatory disease |  | Prednisone 5 mg/day |

*Anonymized identifiers (H = hematological, T = solid organ transplant, S = solid tumor only, O = other)*

Abbreviations: ALL, acute lymphoblastic leukaemia; AML, acute myeloid leukaemia; CRF, case-report form; CT, computed tomography; HSCT, hematopoietic stem cell transplantation; ICU, intensive care unit; L-AmB, liposomal amphotericin B; MDS, myelodysplastic syndrome; MTX, methotrexate; PCR, polymerase chain reaction.
